# Curcumin and piperine in neurodegenerative disorders: a systematic review of preclinical neuroprotective evidence

**DOI:** 10.3389/fnut.2026.1855377

**Published:** 2026-07-09

**Authors:** Minji Zhou, Xiyun Wang

**Affiliations:** 1 Chinese Medicine Hospital of Tiantai County, Taizhou, Zhejiang, China; 2 Department of Neurology, Tiantai People’s Hospital of Zhejiang Province (Tiantai Branch of Zhejiang Provincial People’s Hospital), Taizhou, Zhejiang, China

**Keywords:** Alzheimer’s disease, curcumin, nanoformulations, neuroinflammation, neuroprotection, oxidative stress, piperine, systematic review

## Abstract

**Background:**

Curcumin and piperine are phytochemicals with neuroprotective potential; however, curcumin’s limited bioavailability restricts its therapeutic efficacy. Increasing evidence suggests that their co-administration may provide synergistic neuroprotective benefits. This systematic review evaluates the current preclinical evidence for curcumin-piperine (CUR-PP) combinations in neurodegenerative disorder models.

**Methods:**

We searched electronic databases for studies published between 2010 and 2026. Twenty unique preclinical studies, involving rodent models and neuronal cell cultures, met the eligibility criteria. Due to substantial methodological heterogeneity in study designs, dosing regimens, and outcome measures, a quantitative meta-analysis was not feasible. Findings were synthesized qualitatively, focusing on behavioral, biochemical, and molecular outcomes.

**Results:**

CUR-PP co-administration consistently demonstrated superior neuroprotective efficacy compared to monotherapy. The combination significantly improved cognitive and locomotor performance, mitigated oxidative stress, and modulated neuroinflammatory pathways. Nanoformulations, such as PLGA nanoparticles, further enhanced bioavailability and therapeutic impact across the included studies.

**Conclusion:**

Preclinical data indicate that CUR-PP combinations exert synergistic neuroprotective effects, suggesting they are promising multi-target candidates for neurodegeneration. However, the therapeutic potential is currently limited by a reliance on preclinical models, substantial inter-study heterogeneity, and a total lack of clinical trial data, which prevents the establishment of validated human dosing protocols.

## Graphical abstract

**Figure:**
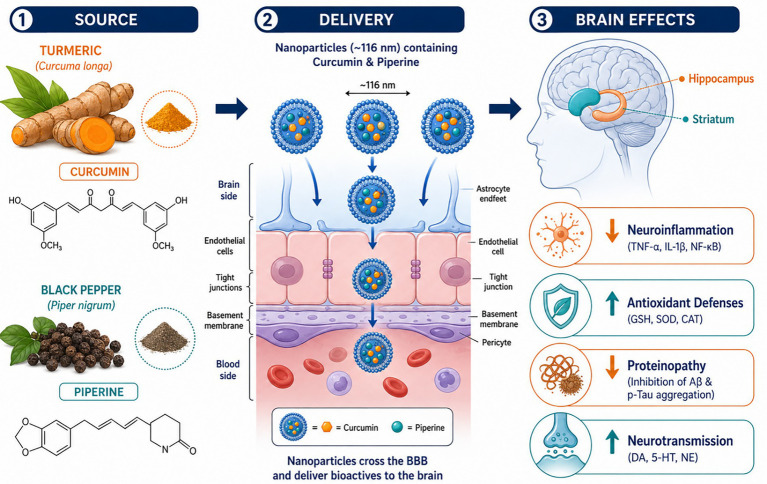
Synergistic neuroprotective effects of curcumin–piperine co-administration on neurotoxicity, cognitive impairment, and neurodegenerative models. The schematic illustrates the multi-targeted mechanism of action and pharmacokinetic enhancement of Curcumin (CUR) when co-administered with Piperine (PP). Pharmacokinetic Potentiation: Piperine acts as a potent bioenhancer by inhibiting hepatic and intestinal glucuronidation, thereby significantly increasing the systemic bioavailability of Curcumin. The incorporation of these compounds into advanced nanoformulations (e.g., PLGA nanoparticles, ∼116 nm) facilitates superior Blood–Brain Barrier (BBB) permeability and targeted delivery to critical neuroanatomical regions, including the hippocampus, striatum, and cerebellum. Mechanistic Synergy in Neuroprotection: The dual-therapy exerts neuroprotective effects through a four-pronged approach: (1) Antioxidant Defense, via the restoration of the endogenous antioxidant pool (↑GSH, SOD, CAT) and suppression of lipid peroxidation (↓MDA, 8-OHdG) and reactive oxygen species (ROS); (2) Anti-inflammatory Signaling, characterized by the downregulation of pro-inflammatory cytokines (TNF-*α*, IL-1β, IL-6) and inhibition of the NF-κB pathway; (3) Anti-apoptotic Pathways, through the attenuation of Caspase-3 and Caspase-9 activation; and (4) Proteinopathy and Neurotransmission Modulation, involving the inhibition of Amyloid-beta (Aβ) aggregation and the restoration of monoaminergic neurotransmitters (Dopamine, Serotonin, Norepinephrine). These molecular shifts culminate in Preserved Functional Outcomes, including HPA-axis normalization (↓serum corticosterone) and significant improvements in cognitive and motor performance across diverse models of Alzheimer’s, Parkinson’s, and Huntington’s disease.

## 1 Introduction

Neurodegenerative disorders, including Alzheimer’s disease (AD), Parkinson’s disease (PD), and age-related cognitive decline, represent major global health challenges with rapidly increasing prevalence due to population aging. According to recent epidemiological estimates, more than 55 million people worldwide are currently living with dementia, and this number is projected to exceed 130 million by 2050. Similarly, Parkinson’s disease prevalence has more than doubled over the past two decades, making neurodegeneration a substantial contributor to disability, healthcare expenditure, and mortality worldwide (1). Despite advances in understanding disease pathogenesis, currently available therapies remain largely symptomatic and provide limited disease-modifying benefit.

The pathophysiology of neurodegenerative disorders is multifactorial and involves interconnected mechanisms including oxidative stress, chronic neuroinflammation, mitochondrial dysfunction, synaptic impairment, and abnormal protein aggregation (2–5). Oxidative stress contributes to neuronal injury through excessive production of reactive oxygen species (ROS), lipid peroxidation, DNA damage, and mitochondrial dysfunction. In parallel, persistent neuroinflammation driven by activated microglia and astrocytes promotes the release of pro-inflammatory cytokines such as TNF-*α*, IL-1β, and IL-6, further exacerbating neuronal degeneration and synaptic dysfunction. Because oxidative and inflammatory pathways interact synergistically during disease progression, multi-target therapeutic strategies capable of modulating both processes simultaneously are increasingly being investigated.

Phytochemicals have attracted considerable attention as potential neuroprotective agents because of their pleiotropic biological activities and relatively favorable safety profiles (6). Curcumin, a polyphenolic compound derived from *Curcuma longa* (turmeric), exhibits potent antioxidant effects through ROS scavenging, enhancement of endogenous antioxidant enzymes, and inhibition of lipid peroxidation. In addition, curcumin exerts anti-inflammatory actions by modulating NF-κB, MAPK, and cytokine signaling pathways, while also demonstrating anti-amyloidogenic and anti-apoptotic properties in experimental models of neurodegeneration (7–10). However, its clinical translation has been hindered by poor oral bioavailability, rapid metabolism, and limited blood–brain barrier (BBB) permeability (5, 11).

Piperine, an alkaloid isolated from *Piper nigrum* (black pepper), has emerged as an important bioenhancer capable of increasing curcumin absorption and systemic availability through inhibition of glucuronidation and modulation of intestinal permeability (2, 7, 12). Beyond its pharmacokinetic effects, piperine also possesses intrinsic antioxidant and anti-inflammatory activities. Experimental studies increasingly suggest that curcumin-piperine (CUR-PP) combinations may exert synergistic neuroprotective effects by simultaneously targeting oxidative stress, neuroinflammation, mitochondrial dysfunction, and protein aggregation pathways (3, 13, 14).

Recent advances in nanotechnology-based delivery systems—including polymeric nanoparticles, self-nanoemulsifying drug delivery systems (SNEDDS), liposomes, and solid-lipid nanoparticles—have further enhanced the therapeutic potential of CUR-PP combinations by improving solubility, stability, BBB penetration, and sustained drug release (5, 11, 15). Nevertheless, concerns remain regarding long-term safety, nanocarrier toxicity, large-scale manufacturing, and translational reproducibility.

Although several narrative reviews have discussed curcumin or phytochemicals in neurodegenerative diseases, few have specifically focused on the synergistic interaction between curcumin and piperine, and fewer still have systematically integrated evidence regarding nanoformulations, mechanistic pathways, behavioral outcomes, and translational limitations across preclinical models. Existing reviews also tend to evaluate curcumin monotherapy without critically examining the role of piperine-mediated bioavailability enhancement or comparing conventional and nanoformulated CUR-PP approaches.

Therefore, the present systematic review aims to comprehensively evaluate preclinical evidence regarding the neuroprotective effects of curcumin, piperine, and their combined or nanoformulated interventions in models of neurodegeneration, neurotoxicity, aging, oxidative stress, and neuroinflammation. Particular emphasis is placed on antioxidant and anti-inflammatory mechanisms, amyloid and tau modulation, mitochondrial protection, apoptotic signaling, neurotransmitter regulation, and bioavailability enhancement strategies. In addition, this review critically examines translational challenges, methodological limitations, and future clinical implications to provide a more integrated and updated perspective on the therapeutic potential of CUR-PP combinations in neurodegenerative disorders.

## 2 Methods

### 2.1 Search strategy

This systematic review was conducted in accordance with the PRISMA 2020 (Preferred Reporting Items for Systematic Reviews and Meta-Analyses) guidelines. A comprehensive literature search was performed to identify preclinical studies investigating the neuroprotective effects of curcumin, piperine, and their combined or nanoformulated interventions.

Electronic databases including PubMed/MEDLINE, Scopus, Web of Science, and ScienceDirect were searched from January 2010 to December 2025. The search strategy utilized a combination of Medical Subject Headings (MeSH) terms and free-text keywords related to the intervention, target conditions, and delivery systems. To ensure reproducibility and optimal sensitivity, the following search string was applied (adapted for database-specific syntax where necessary):

(curcumin OR “turmeric”) AND (piperine OR “bioperine” OR “*piper nigrum*”) AND (“neuroprotect*” OR “neurodegen*” OR “Alzheimer*” OR “Parkinson*” OR “cogniti*” OR “memory” OR “oxidative stress” OR “neuroinflam*”) AND (nanoparticle* OR nanoformulat* OR nanoemuls* OR SNEDDS OR PLGA OR bioavailab*)

Additional studies were identified through manual screening of reference lists from relevant reviews and eligible original studies to ensure completeness. No geographical restrictions were applied. Only studies published in English were included to ensure accurate interpretation of complex methodological and mechanistic data; the potential for language bias is acknowledged as a limitation.

### 2.2 Eligibility criteria

*Inclusion criteria:*

1. Original experimental studies (*in vivo* or *in vitro*).
2. Investigations involving curcumin, piperine, or their combination, including nanoformulations.
3. Use of preclinical models of neurodegeneration, neurotoxicity, oxidative stress, neuroinflammation, aging, or cognitive impairment.
4. Reporting of at least one behavioral, biochemical, neurochemical, molecular, or mechanistic outcome related to neuroprotection.
5. Evaluation of synergistic, additive, or bioavailability-enhancing effects of curcumin-piperine combinations.

*Exclusion criteria:*

1. Clinical trials, case reports, reviews, editorials, or conference abstracts.
2. Lack of neurological, cognitive, or mechanistic endpoints.
3. Investigations of curcumin or piperine in non-neurological disease models.
4. Insufficient methodological or outcome data.
5. Studies unavailable in full text or published in languages other than English.

### 2.3 Study selection process

All retrieved records were imported into reference management software, and duplicate records were removed. Study selection was conducted in two stages by two independent reviewers:

1. *Title and abstract screening*: Titles and abstracts were screened against the eligibility criteria; clearly irrelevant studies were excluded.
2. *Full-text assessment*: Full texts of potentially eligible studies were independently assessed for final inclusion.

**Figure 1:**
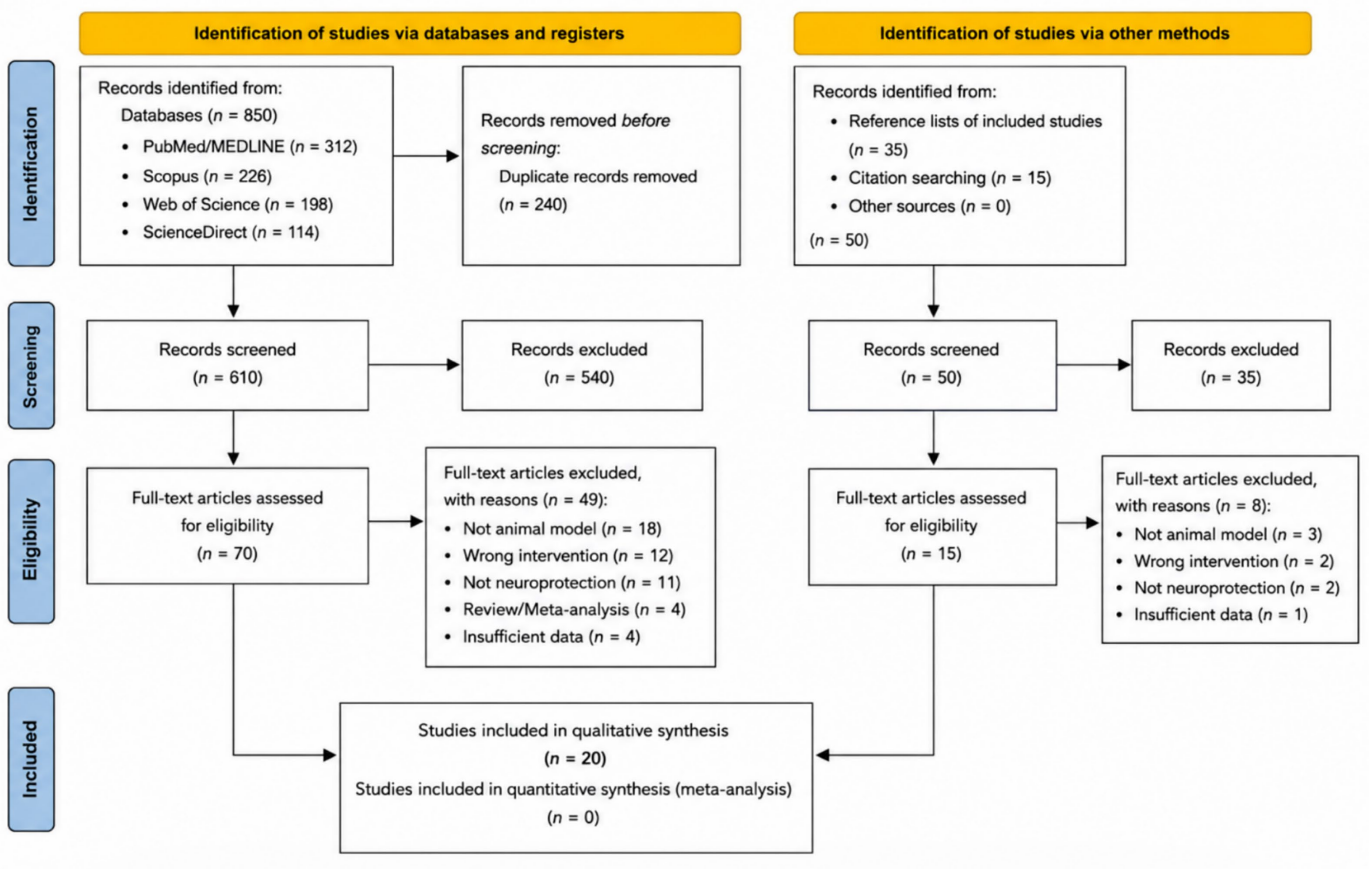
PRISMA flow diagram of study selection. Records were identified through database searching (PubMed, Scopus, Web of Science, Google Scholar) and other sources (reference lists and citation tracking). After screening and eligibility assessment, 20 studies were included in the qualitative synthesis.

Disagreements at both stages were resolved through discussion and consensus. Inter-reviewer agreement was quantified using Cohen’s kappa (*κ*\kappaκ) coefficient. Agreement was substantial (κ > 0.80), indicating high consistency between reviewers. A total of 20 studies met all criteria and were included (Figure 1).

### 2.4 Data extraction and synthesis

Data extraction was independently performed by two reviewers using a standardized form. Extracted information included experimental model/species, intervention type, dose/route/duration, outcomes (behavioral, biochemical, etc.), mechanistic pathways, and evidence of synergy or bioavailability enhancement. Discrepancies were resolved through consensus-based re-evaluation.

A formal quantitative meta-analysis was not feasible due to significant clinical and methodological heterogeneity across the included studies, which would render pooled effect sizes statistically invalid and potentially misleading. The primary sources of this heterogeneity include:

- *Methodological diversity*: studies utilized a broad spectrum of neurodegenerative models, including diverse genetic and chemically induced rodent models (e.g., AlCl3_33-induced, STZ-induced) alongside varied *in vitro* neuronal culture systems.
- *Interventional variability*: There was substantial variation in dosage ranges (Curcumin: 25–400 mg/kg; Piperine: 2.5–60 mg/kg), administration routes (e.g., oral gavage, intraperitoneal, dietary), and duration of treatment (ranging from 7 days to 8 weeks), complicating the determination of a standardized therapeutic regimen.
- *Outcome measure inconsistency*: The included studies employed a wide array of non-standardized behavioral paradigms (e.g., Morris Water Maze, Y-maze, Open Field Test) and biochemical assays with different units of measure and time points for analysis.

Given these disparities, a narrative synthesis was employed to group findings by behavioral, biochemical, and mechanistic outcomes, providing a more reliable and nuanced interpretation of the current preclinical evidence base.

### 2.5 Risk of bias and study quality assessment

Risk of bias for *in vivo* studies was assessed using the SYRCLE (Systematic Review Centre for Laboratory Animal Experimentation) Risk of Bias tool. The tool evaluates 10 domains across six categories: selection bias (sequence generation, baseline characteristics, and allocation concealment), performance bias (random housing and blinding), detection bias (random outcome assessment and blinding), attrition bias, reporting bias, and other potential biases.

*In vitro* studies were evaluated separately, with non-applicable domains designated as N/A. To provide an overall assessment, studies were categorized as low, moderate, or high risk of bias based on the distribution of SYRCLE judgments. Studies with predominantly low-risk ratings were classified as low risk, those with multiple unclear domains as moderate risk, and those with one or more high-risk domains affecting internal validity as high risk.

Assessments were performed independently by two reviewers. Inter-rater reliability for the risk-of-bias assessment was quantified using Cohen’s kappa coefficient, yielding a value of 0.81, which indicates substantial agreement. Any discrepancies between reviewers were resolved through discussion and consensus (Table 1).

**Table 1:** SYRCLE risk of bias summary (included studies, *n =* 20).

Study (author, year)	Selection bias (randomization and allocation)	Performance bias (blinding)	Detection bias (outcome assessment)	Attrition bias	Reporting bias	Other bias	Overall risk
Bishnoi et al., 2010 (12)	Unclear	High	Unclear	Low	Low	Low	High
Rinwa et al., 2012 (2)	Unclear	High	Unclear	Low	Low	Low	High
Banji et al., 2013 (BR) (3)	Unclear	High	Unclear	Low	Low	Low	High
Banji et al., 2013 (EJP) (16)	Unclear	High	Unclear	Low	Low	Low	High
Singh et al., 2015 (7)	Unclear	High	Unclear	Low	Low	Low	High
Singh et al., 2016 (18) (QA)	Unclear	High	Unclear	Low	Low	Low	High
Singh et al., 2016 (17) (6-OHDA)	Unclear	High	Unclear	Low	Low	Low	High
Jangra et al., 2016 (8)	Unclear	High	Unclear	Low	Low	Low	High
Ebrahimnejad et al., 2024 (14)	Unclear	Unclear	Unclear	Low	Low	Low	Moderate
Aygörmez et al., 2024 (10)	Unclear	Unclear	Unclear	Low	Low	Low	Moderate
Alam et al., 2025 (15)	Unclear	Unclear	Unclear	Low	Low	Low	Moderate
Phuna et al., 2025 (5)	Low	Unclear	Unclear	Low	Low	Low	Low-Mod
Saberi-Hasanabadi et al., 2025 (22)	Unclear	Unclear	Unclear	Low	Low	Low	Moderate
Erfen et al., 2022 (19) (cell)	N/A	N/A	Unclear	Low	Low	Low	N/A (In vitro)
Manap et al., 2019 (4)	N/A	N/A	Unclear	Low	Low	Low	N/A (In vitro)
Manap et al., 2020 (13)	N/A	N/A	Unclear	Low	Low	Low	N/A (In vitro)
Sarawi et al., 2021 (9)	Unclear	Unclear	Unclear	Low	Low	Low	Moderate
Ahmad et al., 2023 (11)	Unclear	Unclear	Unclear	Low	Low	Low	Moderate
Rinwa et al., 2012 (7)	Unclear	High	Unclear	Low	Low	Low	High
Alam et al., 2025 (27)	Unclear	Unclear	Unclear	Low	Low	Low	Moderate

## 3 Results

### 3.1 Study selection

A total of 20 preclinical studies published between 2010 and 2025 met the eligibility criteria and were included in the final qualitative synthesis. The study selection process is summarized in Figure 1 (PRISMA flow diagram). All included studies investigated the neuroprotective effects of curcumin (CUR), piperine (PP), or their combined and nanoformulated interventions in experimental models of neurodegeneration, neurotoxicity, oxidative stress, or cognitive impairment.

### 3.2 Study characteristics

The characteristics of the included studies are summarized in Table 2. Most investigations employed *in vivo* rodent models (rats or mice), whereas a smaller number utilized *in vitro* neuronal systems, including SH-SY5Y cells and primary astrocyte cultures.

**Table 2:** Characteristics of included studies (*n =* 20).

Study (author, year)	Model/species	Disease model	Intervention	Dose and duration	Key outcomes	Main findings
Bishnoi et al., 2010 (12)	Rat	Haloperidol neurotoxicity	CUR ± PP	CUR: 25 mg/kg; PP: 2.5 mg/kg; 21 days	Oxidative stress ↓, DA/5-HT/NE ↑	PP enhanced CUR neuroprotection
Rinwa et al., 2012 (2)	Mouse	Chronic stress	CUR + PP	CUR:100 or 200; PP: 20 mg/kg; 28 days	Memory ↑, MDA ↓, GSH ↑	Improved cognition; synergistic effect
Banji et al., 2013 (3)	Rat	D-galactose aging	CUR + PP	CUR: 100 mg/kg; PP: 20 mg/kg; 56 days	Memory ↑, lipid/protein oxidation ↓	Reduced oxidative damage, improved memory
Banji et al., 2013 (16)	Rat	Aging (D-galactose)	CUR + PP	CUR: 40 mg/kg; PP: 12 mg/kg;49 days	Hippocampal neurons ↑, oxidative stress ↓	Enhanced neuroprotection and signaling
Singh et al., 2015 (7)	Rat	3-NP neurotoxicity	CUR ± PP	25–50 mg/kg; 21 days	DA/NE/5-HT ↑, TNF-α ↓, MDA ↓	PP potentiated CUR effects
Singh et al., 2016 (18)	Rat	QA-induced HD model	CUR ± PP	CUR: 25 mg/kg; PP: 2.5 mg/kg;21 days	Behavior ↑, cytokines ↓, GSH ↑	Enhanced antioxidant and neuroprotection
Singh et al., 2016 (17)	Rat	6-OHDA Parkinson model	CUR ± PP	CUR: 25 mg/kg; PP: 2.5 mg/kg; 21 days	Motor ↑, DA ↑, oxidative stress ↓	Improved motor and biochemical outcomes
Jangra et al., 2016 (8)	Mouse	LPS neuroinflammation	CUR ± PP	CUR: 100, 200, 400 mg/kg; PP: 20 mg/kg; 7 days	IL-1β/TNF-α ↓, anxiety ↓	Reduced inflammation; synergistic effect
Ebrahimnejad et al., 2024 (14)	Mouse	Methamphetamine toxicity	CUR-PP NP	NP -CUR: 20, 40, 60 mg/kg; NP -PP: 10, 20, 40 mg/kg	ROS ↓, behavior ↑	Reduced oxidative damage, improved behavior
Aygörmez et al., 2024 (10)	Rat	Cyclophosphamide toxicity	CUR + PP	CUR: 200–300 mg/kg; PP: 200–300 mg;7 days	MDA ↓, IL-6 ↓, SOD/CAT ↑	Reduced inflammation, partial neuroprotection
Alam et al., 2025 (15)	Mouse	Cuprizone neurotoxicity	CUR-PP NP	CUR: 10 mg/kg; PP: 3 mg/kg;7 weeks	Memory ↑, GFAP ↓, cytokines ↓	Improved memory, reduced neuroinflammation
Phuna et al., 2025 (5)	Rat	STZ-induced AD	CUR-PP PLGA NP	CUR: 20 mg/kg; PP: 2 mg/kg; 21–28 days	Memory ↑, neuronal damage ↓	Enhanced cognition and neuronal protection
Saberi-Hasanabadi et al., 2025 (22)	Mouse	Methamphetamine toxicity	CUR-PP NP	CUR-NP: 60 mg/kg; PP-NP: 40 mg/kg	ROS ↓, MWM ↑, GSH ↑	Improved memory, reduced oxidative stress
Erfen et al., 2022 (19)	Cell (astrocyte)	Aluminum toxicity	CUR + PP	μM range	Cell viability ↑, apoptosis ↓, IL-6 ↓	Increased survival, reduced cytokines
Manap et al., 2019 (4)	SH-SY5Y cells	AD (Aβ toxicity)	CUR + PP	CUR:35Μm; PP:35 μM	AChE ↓, Aβ aggregation ↓, viability ↑	Synergistic inhibition of Aβ and AChE
Manap et al., 2020 (13)	SH-SY5Y cells	AD (Aβ pathway)	CUR + PP	CUR:35Μm; PP:35 μM	Amyloid genes ↓/modulated	Modulated amyloidogenic genes
Sarawi et al., 2021 (9)	Rat	Copper toxicity	CUR/Nano-CUR	50 mg/kg;7 days	MDA ↓, TNF-α ↓, BAX ↓, BCL-2 ↑	Reduced inflammation and apoptosis
Ahmad et al., 2023 (11)	Rat	AD model	CUR-PP SNEDDS	CUR: 20–40 mg/kg; PP: 2–5 mg/kg; 28 days	Bioavailability ↑, cognition ↑	Improved CNS delivery
Rinwa et al., 2012 (7)	Mouse	Stress model	CUR + PP	CUR:25 mg/kg; PP:2.5 mg/kg;28 days	Memory ↑, behavior ↑	Improved cognition
Alam et al., 2025 (27)	Mouse	Demyelination	CUR-PP nano	CUR: 10 mg/kg; PP: 3 mg/kg; 7 week	Cognition ↑, myelin damage ↓	Improved neuronal integrity

The experimental models encompassed a broad range of neurological conditions, including Alzheimer’s disease, Parkinson’s disease, Huntington-like neurodegeneration, methamphetamine-induced neurotoxicity, D-galactose-induced aging, chronic unpredictable stress, lipopolysaccharide-induced neuroinflammation, cyclophosphamide-induced neurotoxicity, and metal-induced neurotoxicity.

Considerable methodological heterogeneity was observed among studies regarding animal models, intervention protocols, formulation types, dosing regimens, and treatment durations. Curcumin doses ranged from 10 to 400 mg/kg/day, while piperine doses ranged from 2.5 to 40 mg/kg/day. Treatment duration varied between 7 and 56 days. Several studies incorporated nanoformulations, including PLGA nanoparticles, self-nanoemulsifying drug delivery systems (SNEDDS), and nanoemulsions, which generally reported improved pharmacokinetic and neuroprotective outcomes compared with conventional formulations.

### 3.3 Behavioral and cognitive outcomes

#### 3.3.1 Learning and memory

Most studies reported improvements in learning and memory following CUR-PP administration. Enhanced performance was observed in behavioral paradigms including the Morris Water Maze, Passive Avoidance Test, Elevated Plus Maze, and Open Field Test. Comparative findings suggested that nanoformulated CUR-PP combinations frequently produced greater cognitive improvements than monotherapy or non-nanoformulated preparations, particularly in streptozotocin- and cuprizone-induced models of neurodegeneration.

However, the magnitude of behavioral improvement varied across studies, likely reflecting differences in disease models, treatment duration, and formulation characteristics. In several studies, cognitive improvements were modest despite biochemical changes, highlighting variability in functional responsiveness among experimental systems (16).

#### 3.3.2 Motor function

In toxin-induced models of Parkinsonian and Huntington-like neurodegeneration, including 6-OHDA, quinolinic acid, and 3-nitropropionic acid models, CUR-PP treatment was associated with improved locomotor activity, grip strength, and motor coordination. Studies employing nanoparticle formulations generally demonstrated greater preservation of nigrostriatal integrity and behavioral recovery compared with conventional formulations (17, 18).

#### 3.3.3 Anxiety- and depressive-like behaviors

Several studies reported attenuation of anxiety- and depressive-like behaviors in chronic stress and neuroinflammatory models following CUR-PP treatment. Improvements included enhanced exploratory behavior, reduced anxiety indices, and restoration of sucrose preference. Nevertheless, behavioral outcomes were not uniformly assessed across studies, limiting direct comparison between experimental paradigms.

### 3.4 Biochemical and neurochemical outcomes

#### 3.4.1 Oxidative stress markers

Most studies demonstrated reductions in oxidative stress markers following CUR-PP treatment, including decreases in reactive oxygen species, malondialdehyde, nitrite levels, and protein carbonyl formation, alongside increases in endogenous antioxidant defenses such as glutathione, superoxide dismutase, catalase, and glutathione peroxidase.

Although these findings were generally consistent, the extent of antioxidant effects differed considerably between studies. Nanoformulated CUR-PP preparations often demonstrated greater biochemical efficacy than free compounds, suggesting improved bioavailability and tissue penetration may contribute to enhanced antioxidant activity.

#### 3.4.2 Neuroinflammatory markers

CUR-PP treatment was frequently associated with reduced expression of inflammatory mediators, including TNF-*α*, IL-1β, IL-6, MCP-1, and GFAP, particularly in models of lipopolysaccharide-induced neuroinflammation and cuprizone-induced demyelination. However, inflammatory outcomes varied depending on the experimental model and biomarkers evaluated. While most studies supported anti-inflammatory effects, the mechanistic relationship between cytokine reduction and functional neuroprotection remains incompletely established due to limited mechanistic validation.

#### 3.4.3 Neurotransmitter regulation

Several investigations reported partial normalization of neurotransmitter levels, including dopamine, serotonin, norepinephrine, GABA, and glutamate, following CUR-PP administration. Restoration of neurotransmitter metabolites such as DOPAC, HVA, and 5-HIAA was also observed in selected studies. Nevertheless, neurochemical analyses were performed inconsistently across experiments, limiting broad conclusions regarding neurotransmitter modulation.

### 3.5 Molecular mechanisms

#### 3.5.1 Anti-amyloidogenic and anti-tau effects

In Alzheimer-related models, CUR-PP treatment was associated with reduced amyloid-*β* aggregation and alterations in amyloidogenic signaling pathways. Some studies also reported reductions in phosphorylated tau expression. However, these findings were derived predominantly from preclinical and cell-based experiments, and the precise molecular mechanisms underlying these effects remain incompletely characterized.

#### 3.5.2 Apoptosis and cell survival

Several studies demonstrated modulation of apoptosis-related markers following CUR-PP administration, including reductions in BAX, caspase-3, and p53 expression, together with increased BCL-2 levels. These findings were generally associated with improved neuronal viability and reduced histological damage. Nevertheless, causal relationships between pathway modulation and long-term neuroprotection require further experimental validation.

#### 3.5.3 Signaling pathways

CUR-PP interventions were reported to influence signaling pathways implicated in oxidative stress and inflammation, including Akt/GSK-3β and NF-κB signaling. However, evidence for direct pathway-specific modulation remains preliminary, as most studies relied primarily on biomarker expression rather than comprehensive mechanistic analyses.

### 3.6 Bioavailability enhancement and nanoformulations

Piperine consistently enhanced curcumin bioavailability and appeared to improve neuroprotective efficacy across multiple studies. Nanoformulation strategies—including PLGA nanoparticles, SNEDDS, and nanoemulsions—generally demonstrated superior pharmacokinetic properties, enhanced brain delivery, and improved therapeutic outcomes compared with conventional formulations.

Despite these promising findings, comparative evaluation between nanoformulations remains limited due to substantial variability in nanoparticle composition, particle size, dosing strategies, and experimental design.

### 3.7 Co-administration effects

Across most included studies, CUR-PP combination therapy demonstrated greater efficacy than curcumin or piperine monotherapy in behavioral, biochemical, and molecular outcomes. *In vitro* investigations further suggested potential pharmacological synergy, with reported synergism quotient values greater than 1 in selected experiments. However, the degree of synergy varied among studies, and standardized quantitative assessment of synergistic interactions was limited.

### 3.8 Summary of findings

Collectively, the reviewed studies suggest that CUR-PP combinations may exert neuroprotective effects through modulation of oxidative stress, neuroinflammation, apoptosis, and amyloid-related pathways. Nanoformulation approaches frequently enhanced therapeutic responses, likely through improved bioavailability and CNS delivery. Nevertheless, substantial heterogeneity in experimental design, methodological quality, and outcome assessment limits direct comparison across studies and warrants cautious interpretation of mechanistic and translational conclusions.

### 3.9 Overall risk of bias summary

Methodological quality assessment conducted via the SYRCLE Risk of Bias tool revealed that the included studies exhibited a spectrum of quality ranging from moderate to high risk of bias. A significant portion of the evidence base, particularly studies published between 2010 and 2016, was categorized as High Risk, primarily due to a lack of investigator blinding during behavioral assessments (Performance Bias). Conversely, more recent studies (2021–2025) were generally classified as Moderate Risk, reflecting an improvement in reporting standards, though they remained “unclear” regarding specific randomization and allocation concealment protocols.

Across all included *in vivo* studies, markers of attrition and reporting bias were consistently low, indicating high data transparency and complete outcome reporting. However, critical domains such as random sequence generation and blinding of outcome assessors (Detection Bias) were predominantly insufficiently reported. For the *in vitro* studies (4, 13, 19), selection and performance bias domains were largely non-applicable, though detection bias remained unclear.

## 4 Discussion

**Figure 2:**
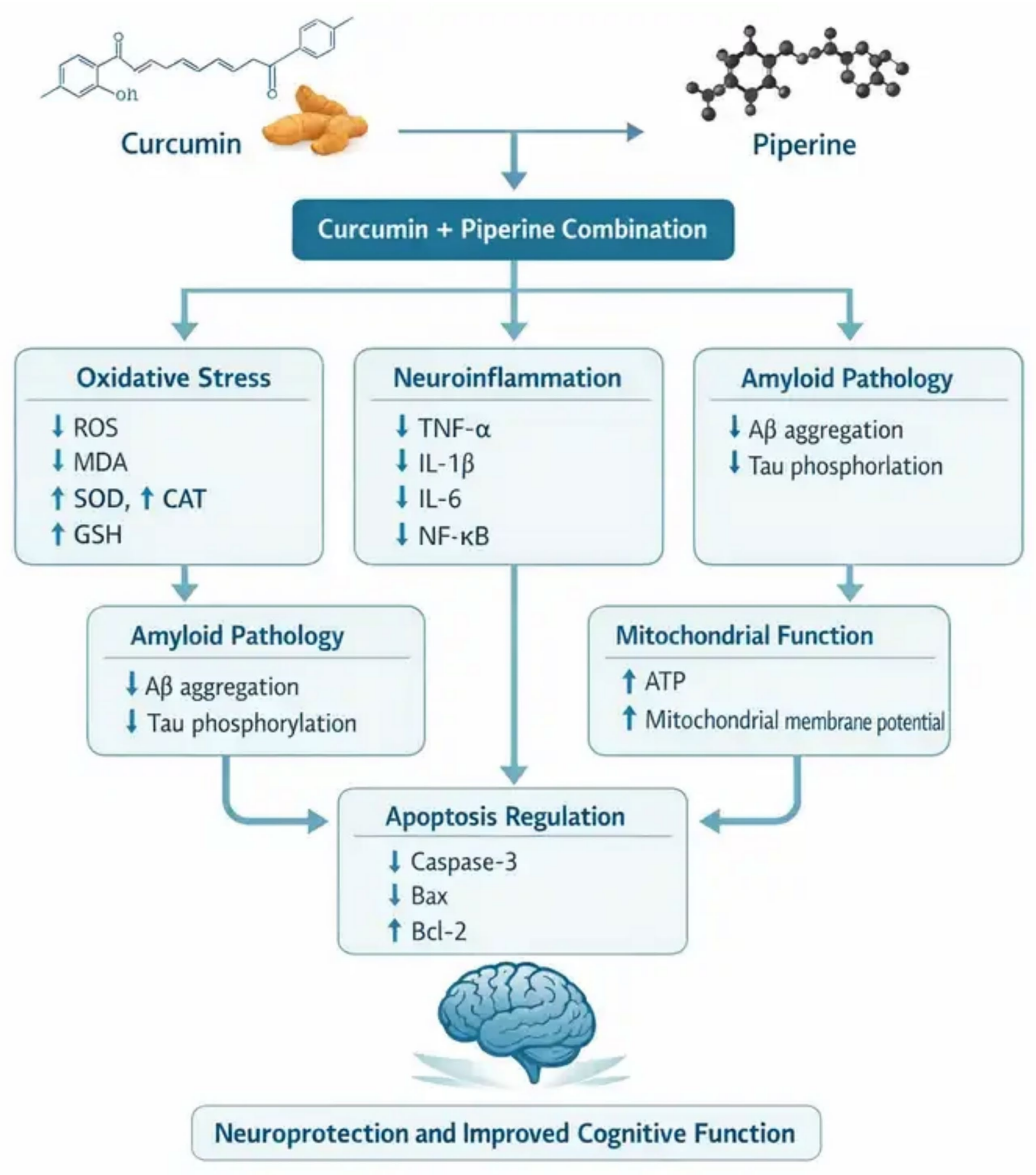
Integrated Molecular Mechanisms of Curcumin and Piperine Synergy in Neuroprotection. This schematic provides a comprehensive overview of the signaling cascades and biochemical pathways modulated by the Curcumin-Piperine (CUR-PP) combination, illustrating how their synergistic interaction targets multiple pathological hallmarks of neurodegeneration to promote neuronal survival and cognitive recovery. Central to this effect is the mitigation of oxidative stress, where the combination restores redox homeostasis by suppressing reactive oxygen species (ROS) and lipid peroxidation (↓MDA), while concurrently upregulating endogenous antioxidant enzymes (↑ SOD, ↑ CAT) and glutathione levels (↑ GSH). Concomitantly, neuroinflammation is attenuated through the inhibition of the NF-*κ* signaling pathway, leading to a significant reduction in the release of pro-inflammatory cytokines, including TNF-*α*\alphaα, IL-1β, and IL-6. The CUR-PP treatment further interferes with amyloid pathology by inhibiting amyloid-beta (Aβ) aggregation and reducing Tau hyperphosphorylation, thereby preventing the formation of neurotoxic plaques and tangles. These protective mechanisms extend to mitochondrial and apoptotic regulation, where enhanced mitochondrial function—evidenced by increased ATP production and stabilization of the mitochondrial membrane potential—converges on the regulation of programmed cell death. This is characterized by the downregulation of pro-apoptotic markers (↓ Caspase-3,↓ Bax) and the upregulation of the anti-apoptotic protein ↑ Bcl-2. Ultimately, the collective modulation of these interconnected pathways results in preserved neuronal architecture, enhanced synaptic plasticity, and significant improvement in cognitive and motor functions across diverse neurodegenerative models.

This systematic review synthesizes current preclinical evidence regarding the neuroprotective potential of curcumin (CUR) and piperine (PP), individually and in combination, across experimental models of neurodegeneration, aging, oxidative stress, neuroinflammation, and chemically induced neurotoxicity. Overall, the reviewed studies indicate that combined CUR–PP interventions produce beneficial effects on cognitive performance, oxidative balance, inflammatory signaling, and neuronal survival, often demonstrating greater efficacy than curcumin monotherapy (2, 4, 5, 7). However, because formal pharmacological synergy analyses were only performed in a limited number of studies, the present review refers primarily to *combined* or *complementary* effects rather than consistently labeling these interactions as synergistic. The inclusion of piperine appears to enhance curcumin bioavailability and central nervous system (CNS) penetration, while nanoformulation strategies further improve pharmacokinetic stability, solubility, and targeted brain delivery (11, 15). As illustrated in Figure 2, CUR and PP influence multiple interconnected pathological pathways, including oxidative stress, neuroinflammation, mitochondrial dysfunction, amyloidogenesis, and apoptosis.

### 4.1 Synergistic cognitive and behavioral effects

Across a broad range of experimental paradigms—including Alzheimer’s disease (AD), Parkinson’s disease (PD), methamphetamine (METH)-induced neurotoxicity, D-galactose-induced aging, and chronic unpredictable stress (CUS)—combined CUR–PP treatment was associated with improvements in learning, memory, motor performance, and affective behaviors (17). These findings support the concept that multi-target phytochemical approaches may provide broader neuroprotective effects than single-target interventions.

In AD-related models, particularly streptozotocin (STZ)-induced neurodegeneration, CUR–PP-loaded PLGA nanoparticles improved performance in Morris Water Maze and Passive Avoidance tasks while preserving hippocampal neuronal architecture (5). Similarly, in amyloid beta (Aβ42)-exposed SH-SY5Y neuronal cells, combined CUR–PP treatment reduced amyloid-associated toxicity and improved cell viability (4, 13). Although these findings are promising, the evidence remains limited to preclinical settings and should not be interpreted as confirmation of clinical efficacy.

**Figure 3:**
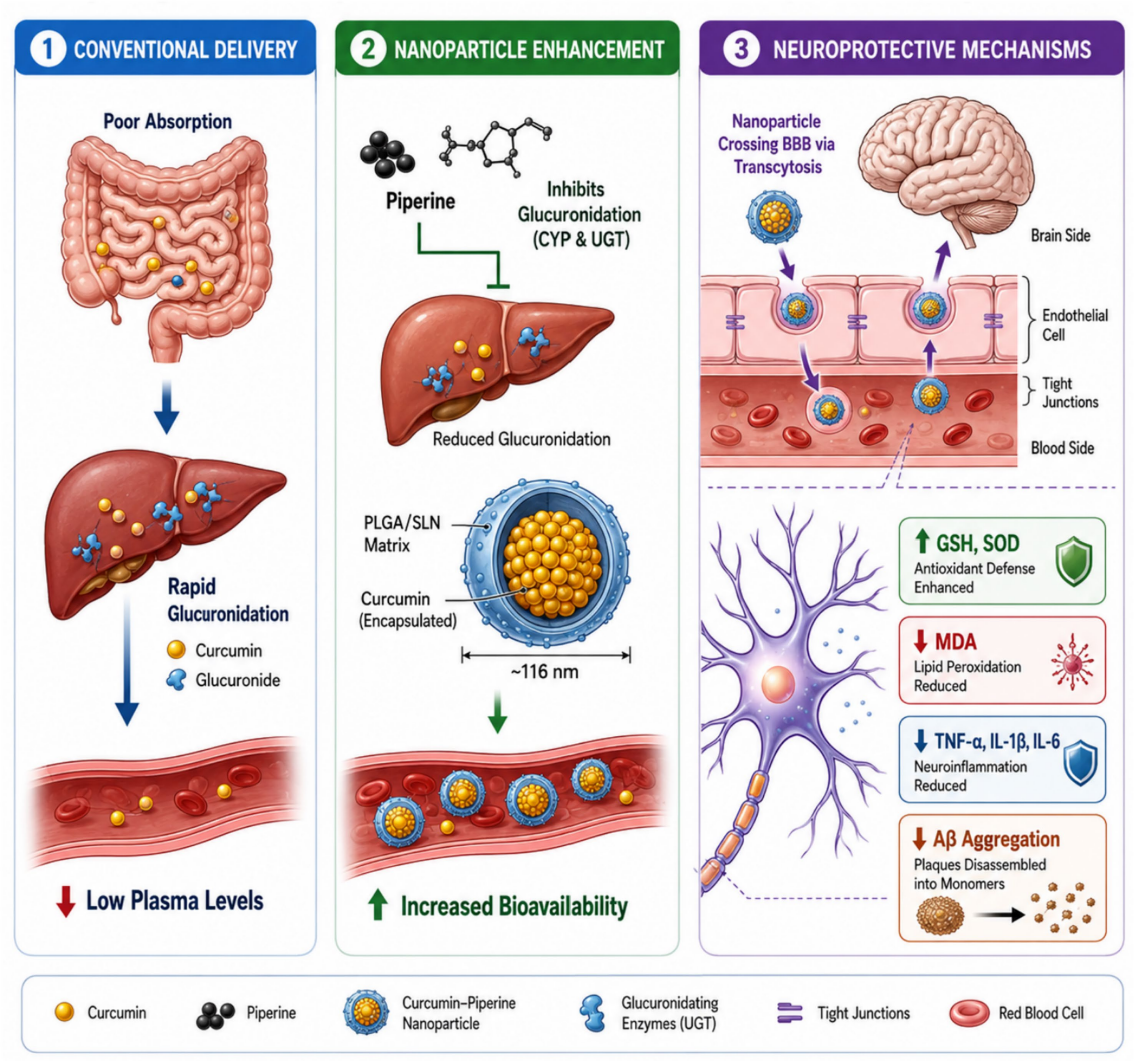
Synergistic Mechanistic Framework for Curcumin-Piperine Nanoformulations in Neuroprotection. This schematic illustrates the pharmacokinetic and pharmacodynamic enhancements achieved through the combined administration of Curcumin (CUR) and Piperine (PP). As depicted in the comparative pharmacokinetics (left), CUR alone is subject to rapid hepatic glucuronidation, resulting in poor systemic bioavailability; however, the co-administration of PP inhibits this metabolic pathway, significantly elevating plasma concentrations and extending half-life. Central to this synergy is the utilization of nanoparticle-mediated delivery systems, such as PLGA (mean diameter ∼116.6 nm) and Solid Lipid Nanoparticles (SLNs), which bypass paracellular restrictions to facilitate active transcellular transport (transcytosis) across the blood–brain barrier (BBB) endothelium. This mechanism ensures superior CNS accumulation compared to unencapsulated compounds. Upon reaching the brain parenchyma, the synergistic pair exerts multimodal neuroprotective effects (right) by restoring antioxidant balance through reduced lipid peroxidation (↓ MDA) and elevated endogenous defenses (↑ GSH, SOD). Furthermore, the formulation suppresses pro-inflammatory signaling (↓ TNF-α\alphaα, IL-1β, IL-6) via NF-κ inhibition, mitigates proteotoxicity by promoting the transition of amyloid-beta (Aβ) fibrils into soluble monomers, and downregulates Caspase-3/9 mediated apoptotic pathways to preserve neuronal integrity and cognitive function.

Aging-related models further demonstrated that CUR–PP administration improved spatial memory, working memory, and sensorimotor coordination, accompanied by preservation of hippocampal CA1 neurons and cerebellar Purkinje cells (3, 16). Improvements in serotoninergic signaling and reductions in oxidative burden were also observed, suggesting that modulation of neurotransmitter homeostasis may contribute to behavioral recovery (Figure 3).

**Figure 4:**
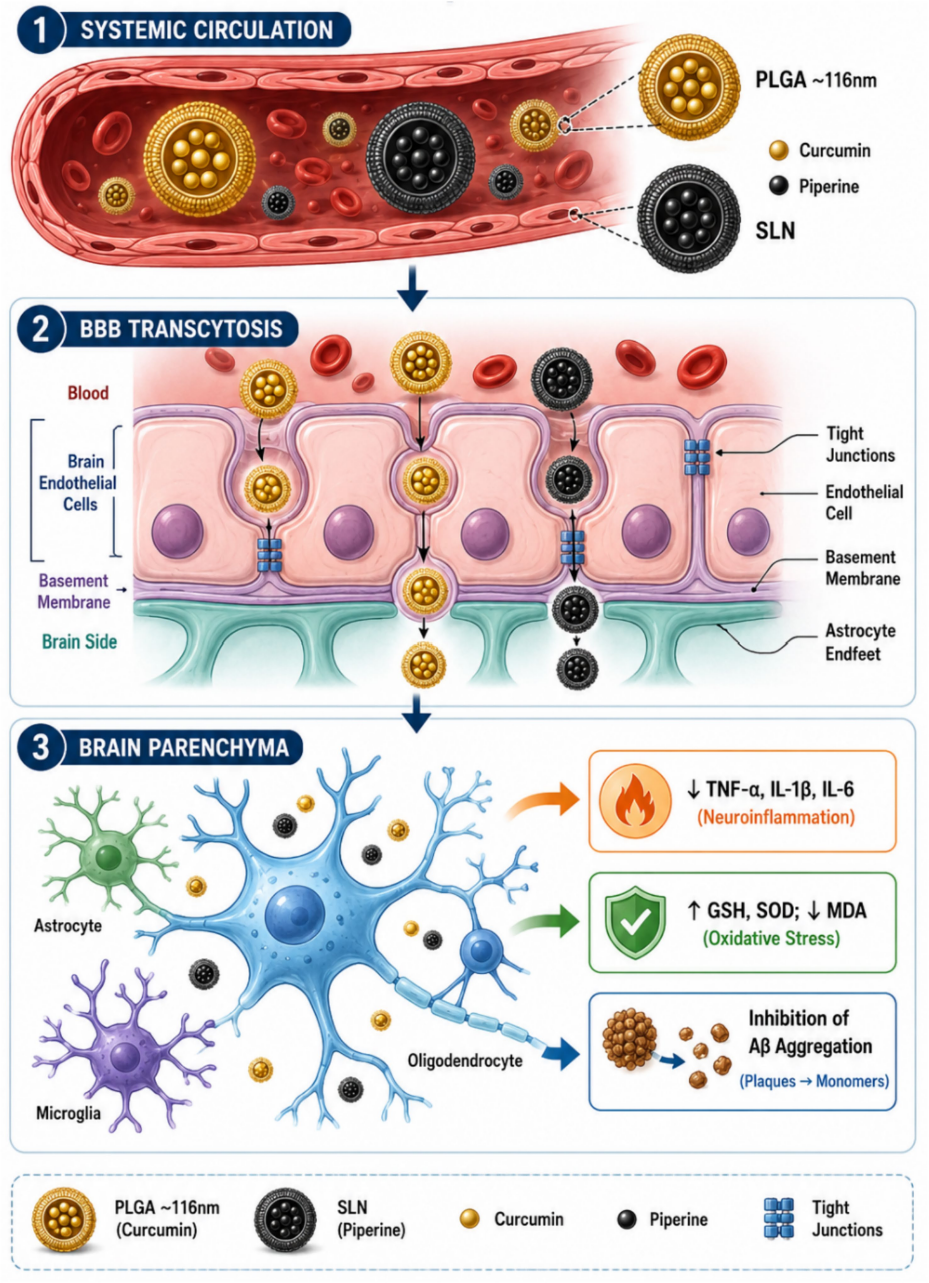
Schematic representation of nanoparticle-mediated delivery and neuroprotective synergy of Curcumin (CUR) and Piperine (PP). The illustration depicts the transition of CUR + PP nanoformulations (including polymeric nanoparticles, solid lipid nanoparticles, and nanoemulsions) from systemic circulation through the blood–brain barrier (BBB). (A) Pharmacokinetic Enhancement: Nano-encapsulation facilitates the circumvention of first-pass metabolism and enhances paracellular or transcellular transport across the BBB endothelium. (B) Parenchymal Interaction: Upon entry into the brain parenchyma, CUR and PP interact with neuronal and glial populations. © Molecular Mechanisms: Synergistic effects are characterized by: (i) ↓ Oxidative Stress: Reduction in reactive oxygen species (ROS) and lipid peroxidation (MDA) with concomitant restoration of glutathione (GSH) and superoxide dismutase (SOD); (ii) ↓ Neuroinflammation: Suppression of the NF-κ pathway and pro-inflammatory cytokines (TNF-α, IL-1β); (iii) ↓ Proteotoxicity: Inhibition of Amyloid-beta (Aβ) fibrillogenesis and plaque aggregation; and (iv) ↓ Apoptosis: Downregulation of Caspase-3/9 mediated programmed cell death. These combined mechanisms preserve neuronal integrity and cognitive/motor function across diverse neurotoxicity models.

The reviewed studies also indicate potential benefits in toxin-induced neurotoxicity. In METH-induced models, CUR–PP nanoparticles attenuated anxiety-like behavior, hyperlocomotion, and conditioned place preference (14). Likewise, in 3-nitropropionic acid (3-NP)- and haloperidol-induced neurotoxicity, CUR–PP treatment improved locomotor activity and exploratory behavior, possibly through preservation of dopaminergic and mitochondrial function (7, 12). In stress-related paradigms, including CUS and lipopolysaccharide (LPS)-induced neuroinflammation, CUR–PP reversed anxiety- and depressive-like behaviors while improving locomotor and cognitive outcomes (2, 8) (Figure 4).

Importantly, recent mechanistic studies outside the included dataset provide additional translational context. Wang et al. (20) demonstrated that restoration of hippocampal synaptic plasticity can ameliorate mood-related deficits in social isolation models, highlighting synaptic preservation as a relevant therapeutic target. Similarly, Luo et al. (21) reported that activation of BDNF–TrkB signaling suppresses neuroinflammation and cognitive impairment in AD models, supporting the broader concept that modulation of neurotrophic signaling pathways may contribute to behavioral improvement.

### 4.2 Oxidative stress modulation

Oxidative stress is widely recognized as a major contributor to neuronal dysfunction and neurodegeneration. Excessive reactive oxygen species (ROS) production promotes lipid peroxidation, mitochondrial impairment, DNA damage, and synaptic dysfunction, ultimately leading to neuronal loss. Across the included studies, CUR–PP treatment consistently reduced oxidative stress markers such as ROS, malondialdehyde (MDA), nitrite levels, and protein carbonyls while restoring endogenous antioxidant defenses, including glutathione (GSH), superoxide dismutase (SOD), catalase (CAT), and glutathione peroxidase (GPx).

For example, Singh et al. (7) demonstrated that CUR–PP administration normalized oxidative damage markers in 3-NP-induced Huntington-like neurotoxicity. Likewise, Ebrahimnejad et al. (14) and Saberi-Hasanabadi et al. (22) reported substantial reductions in ROS production, lipid peroxidation, and mitochondrial dysfunction in methamphetamine-induced neurotoxicity models following CUR–PP nanoparticle treatment. These effects were accompanied by restoration of GSH content and mitochondrial metabolic activity, suggesting preservation of cellular bioenergetics. Aging and metal-induced neurotoxicity models further supported the antioxidant potential of CUR–PP. In D-galactose-induced aging, CUR–PP reduced oxidative burden and preserved neuronal morphology (3).

Similarly, nano-curcumin formulations attenuated copper-induced oxidative stress and inflammatory activation while restoring antioxidant enzyme activity (9). In cyclophosphamide-induced neurotoxicity, combined CUR–PP treatment reduced nitric oxide and MDA levels while enhancing antioxidant defenses (10).

Mechanistically, curcumin acts as a direct ROS scavenger and metal chelator, whereas piperine enhances curcumin absorption and tissue availability. Nanoformulation approaches—including PLGA nanoparticles and self-nanoemulsifying drug delivery systems (SNEDDS)—appear to further improve sustained CNS exposure and antioxidant efficacy (5, 11, 14, 15). Recent translational studies also support the relevance of oxidative stress modulation in neurodegenerative disease. For instance, Cheng et al. (23) demonstrated that inhibition of hippocampal ferroptosis can attenuate diabetic encephalopathy, reinforcing the importance of redox regulation as a therapeutic target in neurological disorders.

### 4.3 Anti-inflammatory and neuroprotective signaling

Chronic neuroinflammation contributes substantially to neurodegenerative progression through sustained activation of microglia and astrocytes and the release of pro-inflammatory mediators such as TNF-*α*, IL-1β, IL-6, MCP-1, and GFAP. Persistent inflammatory activation further amplifies oxidative stress and neuronal dysfunction.

The reviewed evidence indicates that CUR–PP interventions reduce inflammatory signaling across multiple experimental models. In LPS-induced neuroinflammation, CUR–PP reduced hippocampal TNF-α and IL-1β levels while improving behavioral outcomes (8). In cuprizone-induced demyelination, nanoformulated CUR–PP decreased inflammatory chemokines and astrocytic activation markers while restoring antioxidant enzyme activity (15).

Mechanistically, CUR–PP appears to modulate several signaling pathways involved in inflammatory regulation, including NF-κB, MAPK, and JAK/STAT pathways. In cyclophosphamide-induced neurotoxicity, CUR–PP suppressed NF-κB activation and reduced inflammatory cytokine production while enhancing antioxidant defenses (10). Similarly, in aluminum-exposed astrocytes, CUR–PP reduced IL-6 and TGF-*β* release while improving cellular viability (19).

Emerging mechanistic studies provide additional support for these pathways. Ghaffari et al. (24) demonstrated that curcumin sustained-release systems targeting the NLRP3 inflammasome promoted neurological recovery and neurogenesis in spinal cord injury models, highlighting the translational importance of inflammasome modulation. Furthermore, Hui et al. (25) emphasized the therapeutic relevance of natural compounds capable of regulating neuroinflammatory and apoptotic signaling in nervous system disorders. Collectively, these findings reinforce the concept that multi-pathway anti-inflammatory modulation may represent a promising strategy for neuroprotection.

### 4.4 Anti-amyloidogenic and anti-apoptotic effects

Amyloid-beta accumulation, tau hyperphosphorylation, mitochondrial dysfunction, and apoptosis are key pathological processes in AD and related neurodegenerative disorders. Several reviewed studies demonstrated that CUR–PP interventions reduce amyloid-associated toxicity and modulate apoptotic signaling pathways. In SH-SY5Y neuronal models, CUR–PP inhibited Aβ42 aggregation and reduced amyloid-induced oxidative stress (4, 13). Gene expression analyses further suggested modulation of pathways associated with amyloid processing, autophagy, and tau phosphorylation, including GSK3B-related signaling (13).

CUR–PP also reduced apoptotic signaling across several experimental paradigms. Studies reported decreased caspase-3 activation, cytochrome c release, DNA fragmentation, and Bax/Bcl-2 ratio following CUR–PP treatment in models of methamphetamine toxicity, aging, and chemically induced neurodegeneration (3, 9, 10, 14). These effects were associated with preservation of mitochondrial membrane potential and modulation of Akt/GSK-3β signaling.

Recent mechanistic investigations in related phytotherapeutic interventions further support the relevance of anti-apoptotic regulation. Cheng et al. (26) reported that multi-target botanical therapies may suppress apoptosis through coordinated transcriptomic and signaling pathway modulation in intracerebral hemorrhage models. Although these findings were not specific to CUR–PP, they strengthen the broader rationale for investigating multi-component neuroprotective strategies.

### 4.5 Bioavailability enhancement and nanotechnology approaches

A primary obstacle in the clinical translation of curcumin is its poor oral bioavailability, characterized by low aqueous solubility, rapid systemic clearance, and limited penetration of the blood–brain barrier (BBB). Historically, the synergistic role of piperine has been attributed primarily to its inhibition of hepatic and intestinal glucuronidation—the process by which curcumin is conjugated with glucuronic acid to become water-soluble and easily excreted (2, 7).

However, emerging evidence suggests a more complex, multi-modal mechanism of action for piperine. Beyond the inhibition of UDP-glucuronosyltransferase (UGT), piperine acts as a potent modulator of P-glycoprotein (P-gp) and Breast Cancer Resistance Protein (BCRP) efflux pumps. These transporters typically act as biological “gatekeepers,” actively pumping curcumin back into the intestinal lumen; piperine-mediated inhibition of these pumps significantly increases net intestinal absorption. Furthermore, piperine has been shown to enhance membrane fluidity and increase local blood flow to the gastrointestinal tract, facilitating passive diffusion. Across the reviewed literature, this co-administration consistently enhanced the biological activity of curcumin, reinforcing piperine’s role as a critical pharmacokinetic potentiator.

Nanotechnology-based delivery systems provide a parallel solution to these pharmacokinetic hurdles by improving CNS targeting and stability. The implementation of Poly(lactic-co-glycolic acid) (PLGA) nanoparticles, Self-Nanoemulsifying Drug Delivery Systems (SNEDDS), nanoemulsions, and solid lipid nanoparticles has demonstrated superior solubility and sustained release kinetics (5, 11, 15). For instance, Phuna et al. (5) demonstrated that PLGA-curcumin-piperine nanoparticles significantly improved cognitive outcomes and attenuated neuronal pyknosis in STZ-induced Alzheimer’s models, outperforming free compound mixtures.

Similarly, the use of SNEDDS formulated in *Zanthoxylum rhetsa* seed oil (15) exhibited profound protective effects in cuprizone-induced demyelination models by suppressing inflammatory chemokines (MCP-1, MIP-1) and preserving hippocampal integrity. These findings indicate that the integration of piperine with advanced nano-scaffolds addresses the historical pharmacokinetic limitations of curcumin.

Despite these technological strides, translational challenges persist. Future research must address long-term toxicity profiles, the scalability of nano-manufacturing, and the standardization of regulatory approval pathways for “nano-nutraceuticals.” To facilitate clinical feasibility, subsequent studies should prioritize the integration of pharmacodynamic biomarkers, quantitative BBB penetration analysis, and rigorous safety profiling to establish standardized clinical dosing strategies.

### 4.6 Translational implications

The extensive preclinical evidence reviewed underscores the therapeutic potential of curcumin-piperine (CUR-PP) combinations as multi-target neuroprotective agents. Neurodegenerative diseases, including AD, PD, and aging-related cognitive decline, are multifactorial disorders characterized by oxidative stress, mitochondrial dysfunction, neuroinflammation, protein aggregation, and neurotransmitter imbalance. CUR-PP acts on all of these pathological pathways simultaneously, which is a critical advantage over conventional single-target therapies (5, 13, 16, 17).

From a translational perspective, the combined effects of CUR-PP are particularly noteworthy. Piperine enhances curcumin’s oral bioavailability and CNS penetration, while nanoformulations—such as PLGA nanoparticles, SNEDDS, and solid lipid nanoparticles—further optimize brain delivery, improve pharmacokinetics, and enable sustained drug release (5, 11, 15). These strategies overcome major limitations of curcumin therapy, facilitating clinically relevant tissue concentrations capable of modulating oxidative stress markers (ROS, MDA, GSH), inflammatory cytokines (TNF-*α*, IL-6, IL-1β), amyloid-beta aggregation, tau phosphorylation, and apoptotic signaling (9, 10).

Moreover, CUR-PP demonstrates efficacy across a wide spectrum of preclinical models, including chemically induced neurotoxicity (methamphetamine, 3-NP, cyclophosphamide, copper, and cuprizone), aging models (D-galactose, senescence), and chronic stress paradigms (2, 7, 14). This versatility suggests potential generalizability to multiple neurodegenerative conditions, not limited to a single pathology. Behavioral improvements—such as enhanced learning, memory, and locomotor activity—paired with molecular and histological neuroprotection, highlight CUR-PP as a holistic neurotherapeutic candidate.

The adjunctive use of CUR-PP with existing pharmacotherapies could address unmet clinical needs. For instance, in AD, where current acetylcholinesterase inhibitors provide only symptomatic relief, CUR-PP could complement treatment by targeting underlying oxidative and amyloidogenic pathology (4). Similarly, in PD or chemotherapy-induced neurotoxicity, CUR-PP could mitigate mitochondrial dysfunction and neurotransmitter alterations, potentially reducing disease progression or drug-related side effects (10, 12).

Finally, the incorporation of CUR-PP into nanomedicine platforms offers opportunities for clinical translation. Nanoparticle delivery enhances solubility, stability, and BBB permeability, allowing oral or parenteral administration with predictable pharmacokinetics, which is essential for patient adherence and long-term therapy. Considering the favorable safety profile of both curcumin and piperine, along with their demonstrated efficacy in preclinical models, CUR-PP holds significant promise for future clinical trials aimed at slowing neurodegeneration, improving cognitive outcomes, and providing neuroprotection across diverse patient populations.

In summary, CUR-PP represents a multi-modal, synergistic, and translationally viable neurotherapeutic strategy, combining molecular, cellular, and behavioral benefits with enhanced bioavailability and CNS delivery, and may form the basis for next-generation adjunctive therapies for neurodegenerative diseases.

### 4.7 Limitations

Despite the substantial preclinical evidence supporting the neuroprotective and combined effects of curcumin-piperine (CUR-PP) combinations, several limitations must be acknowledged before translating these findings to clinical applications.

#### 4.7.1 Predominance of preclinical models

All 20 studies reviewed are preclinical, conducted in rodent models (rats or mice) or *in vitro* neuronal cultures (SH-SY5Y, astrocytes). While these models provide valuable mechanistic insights, they may not fully recapitulate the complex pathophysiology of human neurodegenerative diseases, including Alzheimer’s disease, Parkinson’s disease, and aging-related cognitive decline. Species-specific differences in drug metabolism, blood–brain barrier permeability, immune responses, and neuronal network organization may significantly influence translational efficacy and therapeutic outcomes in humans.

#### 4.7.2 Dose standardization and treatment duration

CUR-PP studies vary considerably in dosage, route of administration, formulation type, and treatment duration (e.g., 25–400 mg/kg curcumin, 2.5–60 mg/kg piperine, oral vs. intraperitoneal delivery). Such heterogeneity complicates comparison across studies and limits determination of clinically relevant dosing strategies. Future investigations should prioritize standardized experimental protocols, dose–response studies, and pharmacokinetic profiling to establish safe and effective therapeutic regimens for human use.

#### 4.7.3 Limited long-term safety data

Most studies evaluated short- to medium-term interventions, typically ranging from 7 days to 8 weeks. Consequently, long-term safety, tolerability, and potential drug–drug interactions remain insufficiently explored, particularly in elderly patients who commonly receive multiple medications. Chronic exposure studies in aged animal models and carefully monitored clinical trials are necessary to evaluate sustained efficacy, cumulative toxicity, and metabolic consequences associated with prolonged CUR-PP administration.

#### 4.7.4 Potential publication bias and selective reporting bias

The reviewed literature may be affected by publication bias, as studies reporting positive neuroprotective outcomes are more likely to be published than studies with negative or inconclusive findings. Additionally, selective reporting of favorable biochemical or behavioral endpoints may overestimate the therapeutic potential of CUR-PP combinations. The limited availability of unpublished data and inconsistent reporting of experimental methodologies further restrict objective assessment of reproducibility and overall evidence quality.

#### 4.7.5 Lack of quantitative meta-analysis

Although this review summarizes current evidence comprehensively, it does not include a formal meta-analysis. Due to substantial heterogeneity in animal models, outcome measures, formulations, dosing regimens, and study designs, quantitative synthesis was not feasible. As a result, pooled effect sizes, statistical comparisons, and assessments of inter-study variability could not be performed. Future systematic reviews incorporating standardized methodologies and larger datasets may allow robust meta-analytic evaluation of CUR-PP efficacy and safety.

#### 4.7.6 Incomplete mechanistic characterization

Although CUR-PP combinations have demonstrated modulation of oxidative stress, neuroinflammation, amyloidogenic pathways, and mitochondrial dysfunction, the precise molecular targets and signaling cascades remain incompletely understood. Potential interactions with epigenetic regulators, autophagy pathways, synaptic plasticity mechanisms, and gut-brain axis signaling require further investigation. Integration of transcriptomics, proteomics, metabolomics, and systems biology approaches may provide a more comprehensive understanding of CUR-PP-mediated neuroprotection.

#### 4.7.7 Nanotechnology translation challenges and potential toxicity

Nanoformulations such as PLGA nanoparticles, SNEDDS, liposomes, and solid-lipid carriers significantly improve curcumin bioavailability, blood–brain barrier penetration, and sustained release. However, important translational concerns remain unresolved, including large-scale manufacturing, reproducibility, physicochemical stability, regulatory approval, and cost-effectiveness. Furthermore, the potential chronic toxicity of nanocarriers requires greater attention. Long-term accumulation of nanoparticles in neural or peripheral tissues may induce oxidative stress, immune activation, organ toxicity, or altered biodistribution profiles. Comprehensive toxicological studies assessing nanoparticle clearance, biodegradation, immunogenicity, and long-term biocompatibility are therefore essential prior to clinical application.

### 4.8 Future perspectives

#### 4.8.1 Clinical trials and biomarker-driven evaluation

The most urgent next step is the development of well-controlled, randomized, double-blind clinical trials to evaluate the safety, pharmacokinetics, tolerability, and therapeutic efficacy of curcumin-piperine (CUR-PP) combinations in patients with mild cognitive impairment, early Alzheimer’s disease, Parkinson’s disease, and related neurodegenerative disorders. Future trials should incorporate clearly defined clinical endpoints, including cognitive performance scales such as the Mini-Mental State Examination (MMSE), Montreal Cognitive Assessment (MoCA), and Alzheimer’s Disease Assessment Scale–Cognitive Subscale (ADAS-Cog), alongside assessments of quality of life and functional capacity.

In addition, biomarker-driven evaluation should be emphasized to improve translational relevance and mechanistic understanding. Recommended biomarkers include oxidative stress markers (MDA, GSH, SOD, CAT), inflammatory mediators (TNF-*α*, IL-1*β*, IL-6, NF-κB activity), and neurodegeneration-associated biomarkers such as amyloid-β (Aβ42), phosphorylated tau, total tau, and α-synuclein. Neuroimaging endpoints, including MRI-based hippocampal volumetry and PET amyloid/tau imaging, may further strengthen clinical assessment of therapeutic response and disease progression.

#### 4.8.2 Personalized medicine approaches

Considering interindividual variability in bioavailability, metabolism, gut microbiota composition, genetic polymorphisms, and disease heterogeneity, CUR-PP therapies may benefit from a personalized medicine approach. Tailoring formulation type, dosing strategies, and treatment duration according to patient-specific pharmacogenomic and metabolic profiles could optimize therapeutic efficacy while minimizing adverse effects.

#### 4.8.3 Combination and adjunctive therapies

CUR-PP may act synergistically with current standard-of-care pharmacotherapies, including acetylcholinesterase inhibitors, NMDA receptor antagonists, and dopaminergic agents. Future studies should systematically investigate adjunctive treatment regimens to determine whether CUR-PP can enhance therapeutic outcomes, reduce required drug dosages, delay disease progression, or mitigate adverse drug effects in neurodegenerative disorders.

#### 4.8.4 Expanded mechanistic and translational studies

Further research is required to elucidate the effects of CUR-PP on neurogenesis, synaptic plasticity, mitochondrial bioenergetics, autophagy, gut-brain axis communication, and epigenetic regulation. Advanced experimental platforms—including brain organoids, induced pluripotent stem cell-derived neuronal models, multicellular co-culture systems, and blood–brain barrier microfluidic models—could provide more physiologically relevant insights into CUR-PP-mediated neuroprotection. Integration of transcriptomics, proteomics, metabolomics, and systems biology approaches may further clarify molecular targets and signaling networks.

#### 4.8.5 Long-term safety and pharmacological evaluation

Extended-duration studies in aged and comorbid animal models are essential to determine chronic toxicity, cumulative tissue accumulation, immunogenicity, metabolic effects, and potential drug–drug interactions associated with prolonged CUR-PP administration. Such investigations will provide critical pharmacological and toxicological data necessary for bridging preclinical findings to human clinical trials.

#### 4.8.6 Regulatory, manufacturing, and nanoformulation translation

Although nanoformulation strategies such as PLGA nanoparticles, self-nanoemulsifying drug delivery systems (SNEDDS), liposomes, and solid-lipid carriers significantly improve curcumin bioavailability and blood–brain barrier penetration, important translational challenges remain. Future development should prioritize Good Manufacturing Practice (GMP)-compliant scalable production methods capable of ensuring batch-to-batch consistency, physicochemical stability, reproducibility, sterility, and cost-effectiveness.

Regulatory evaluation of CUR-PP nanoformulations will require rigorous characterization of particle size distribution, encapsulation efficiency, release kinetics, biodistribution, biodegradation, nanocarrier clearance, and long-term biocompatibility. Harmonization of nanomedicine regulatory frameworks and establishment of standardized safety assessment guidelines will be essential for accelerating clinical translation and commercial development of CUR-PP-based neuroprotective therapies.

## 5 Conclusion

Current preclinical evidence suggests that the combination of curcumin and piperine (CUR-PP) exerts synergistic neuroprotective effects across multiple experimental models of Alzheimer’s disease, Parkinson’s disease, aging-related cognitive decline, and chemically induced neurotoxicity. The reviewed studies indicate that CUR-PP may modulate several pathological processes associated with neurodegeneration, including oxidative stress, neuroinflammation, amyloid and tau pathology, mitochondrial dysfunction, and neuronal apoptosis. Improvements in cognitive, behavioral, biochemical, and molecular outcomes were consistently observed in both *in vivo* and *in vitro* models.

Piperine-mediated enhancement of curcumin bioavailability, together with nanotechnology-based delivery systems such as PLGA nanoparticles and self-nanoemulsifying formulations, further improved CNS penetration and therapeutic activity in preclinical studies. These findings highlight the potential translational relevance of CUR-PP as a multi-target neuroprotective strategy.

However, interpretation of these findings should remain cautious because the current evidence is limited predominantly to preclinical investigations, with substantial heterogeneity in experimental models, formulations, dosing regimens, and outcome measures. In addition, long-term safety, pharmacokinetics, chronic nanocarrier toxicity, and clinical efficacy in humans remain insufficiently established. Therefore, well-designed translational studies and biomarker-driven clinical trials are essential to validate the therapeutic potential, safety, and clinical applicability of CUR-PP formulations in neurodegenerative disorders.

## Data Availability

The original contributions presented in the study are included in the article/supplementary material, further inquiries can be directed to the corresponding author.
